# Early menopause is associated with abnormal diastolic function and poor clinical outcomes in women with suspected angina

**DOI:** 10.1038/s41598-024-57058-2

**Published:** 2024-03-15

**Authors:** SungA Bae, Seong-Mi Park, So Ree Kim, Mi-Na Kim, Dong-Hyuk Cho, Hee-Dong Kim, Hyun Ju Yoon, Myung-A Kim, Hack-Lyoung Kim, Kyung-Soon Hong, Mi-Seung Shin, Jin-Ok Jeong, Wan-Joo Shim

**Affiliations:** 1 Department of Cardiology, Yongin Severance Hospital, Yonsei University College of Medicine, Seoul, Gyeonggi-Do Republic of Korea; 2 Division of Cardiology, Department of Internal Medicine, Korea University Anam Hospital, Goryeodae-Ro 73, Seongbuk-Gu, Seoul, 02841 Republic of Korea; 3 Division of Cardiology, Department of Internal Medicine, Soonchunhyang University Cheonan Hospital, Cheonan, Republic of Korea; 4 Department of Cardiology, Chonnam National University Hospital, Gwangju, Republic of Korea; 5 Department of Cardiology, Seoul National University Boramae Medical Center, Seoul, Republic of Korea; 6 Department of Cardiology, Hallym University Medical Center, Seoul, Chuncheon, Republic of Korea; 7 Department of Cardiology, Gachon University Gil Medical Center, Incheon, Republic of Korea; 8 Department of Cardiology, Chungnam National University Hospital, Daejeon, Republic of Korea

**Keywords:** Cardiology, Menopause, Risk factors

## Abstract

Early identification of women at high risk for cardiovascular diseases (CVD), with subsequent monitoring, will allow for improved clinical outcomes and generally better quality of life. This study aimed to identify the associations between early menopause, abnormal diastolic function, and clinical outcomes. This retrospective study included 795 menopausal women from is a nationwide, multicenter, registry of patients with suspected angina visiting outpatient clinic. The patients into two groups: early and normal menopause (menopausal age ≤ 45 and > 45 years, respectively). If participants met > 50% of the diastolic function criteria, they were classified as having normal diastolic function. Multivariable-adjusted Cox models were used to test associations between menopausal age and clinical outcomes including the incidence of major adverse cardiovascular events (MACE), over a median follow-up period of 771 days. Early menopause was associated with increased waist circumference (*p* = 0.001), diabetes prevalence (*p* = 0.003), obstructive coronary artery disease (*p* = 0.005), abnormal diastolic function (*p* = 0.003) and greater incidences of MACE, acute coronary syndrome, and hospitalization for heart failure. In patients with abnormal diastolic function, early menopause increased MACE risk significantly, with no significant difference in normal diastolic function. These findings highlight early menopause and abnormal diastolic function as being potential risk markers in women for midlife CVD events.

## Introduction

The risk of cardiovascular disease (CVD) increases with age, and since women generally live longer than men, more women live with and die from CVD^1^. Both early identification of women at high risk for CVD and timely implementation of appropriate lifestyle or therapeutic interventions are crucial for public health.

The risk of CVD in women is considerably lower than that in men at younger ages, but it increases significantly after menopause, bringing their risk more in line with that of men^2^. Natural menopause occurs approximately in the age range of 49–52 years, whereas early menopause is defined as that affecting women aged 40–45 years^3–5^. Women experiencing early menopause may face a higher risk of adverse outcomes and live more years with this increased risk^6–8^. This highlights the importance of evaluating menopausal onset as a CVD risk factor. Menopause may be an indicator not only of reproductive aging but also of overall health and physical aging^9^. Previous research has observed a more pronounced increase in left ventricular (LV) diastolic dysfunction among healthy middle-aged women than in men, suggesting that menopause could accelerate the progression of abnormal diastolic function as women age^10^. In particular, heart failure (HF) with preserved ejection fraction (HFpEF) occurs almost twice as often in women than in men; however, the underlying mechanisms for these differences are not well understood^11^. The Multi-Ethnic Study of Atherosclerosis (MESA) has significantly enhanced our understanding of the pathophysiology associated with subclinical cardiovascular diseases, pinpointing risk factors that evolve into overt cardiovascular disorders and highlighting the critical role of the menopause transition for early cardiovascular preventive measures^12^. However, the precise mechanisms through which early menopause and abnormal diastolic function impact clinical outcomes remain to be fully determined, necessitating additional research to clarify their overall influence on the cardiovascular health of menopausal women. Therefore, our study aimed to investigate the relationship between early menopause, abnormal diastolic function, and major adverse cardiovascular events (MACE) in women with suspected angina.

## Methods

### Study population

The Korean Women’s Chest Pain Registry (KoROSE) is a nationwide, multicenter, prospective study evaluating patients with suspected angina who visit an outpatient clinic. We accessed the KoROSE database using an electronic case report form. Patients from 25 tertiary medical centers were registered between January 2012 and May 2018. Patients who visited the hospital with suspected angina, were aged ≥ 20 years, and underwent invasive coronary angiography (CAG) for coronary artery disease (CAD) detection were enrolled in this study. Patients with end-stage renal disease, malignancy, primary pulmonary hypertension, chronic obstructive pulmonary disease, or an autoimmune disease were excluded from the study. We classified the patients into two groups according to their menopausal age: those with early menopause (menopausal age ≤ 45 years) and normal menopause (menopause age > 45 years). This study protocol was approved by the Institutional Review Boards of all participating centers (Institutional Review Board of Korea University Anam Hospital [Seoul, Korea] number: 2012AN0011). The study protocol conforms to the ethical guidelines of the 1975 Declaration of Helsinki as reflected in a priori approval by the institution's human research committee. All patients provided informed consent.

### Clinical and laboratory assessments

The patients’ demographic and clinical characteristics were obtained from the database. The following characteristics were extracted: age, body mass index, waist circumference, systolic and diastolic blood pressure, heart rate, medication use, and traditional cardiovascular risk factors, including a history of hypertension, diabetes mellitus (DM), dyslipidemia, and current smoking. Detailed chest pain questionnaires were answered by all patients (Supplementary Fig. 1). Hypertension was defined as a history of hypertension or the use of antihypertensive medications. DM was defined as a history of DM or the use of antidiabetic medications. Dyslipidemia was defined as a history of dyslipidemia or the use of anti-dyslipidemia medications. We collected patients' reproductive history via a validated questionnaire at initial CAG admission, focusing on menopausal status, determined by the absence of menstruation for 12 months or surgical history, and HRT use. Menopausal status was analyzed both categorically (premenopausal, postmenopausal). Additionally, we calculated the reproductive lifespan as the difference between menopausal age and menarche.

### Invasive CAG and echocardiography

The degree of epicardial coronary artery stenosis was assessed using CAG. Obstructive CAD was defined as ≥ 50% stenosis of one or more major epicardial coronary arteries. All management strategies for CAD, including coronary revascularization and medications, were chosen at the discretion of the attending physicians following CAG.

Comprehensive transthoracic echocardiography (TTE) was performed before CAG. Echocardiography was performed using either a General Electric Vivid E9 (Horten, Norway) or Phillips IE33 machine (Andover, Massachusetts, USA) with tissue Doppler imaging software and a 2.5–5 MHz variable-frequency, phased array transthoracic transducer. All index TTEs were recorded during routine clinical practice in accordance with the current ASE/EACVI recommendations^13^. Hypertrophic alteration of the LV structure was quantified based on the left ventricular mass index (LVMI) and relative wall thickness (RWT)^14^. An abnormal LV geometry was defined as the composite of concentric remodeling (normal LVMI and increased RWT), eccentric hypertrophy (increased LVMI and normal RWT), and concentric hypertrophy (increased LVMI and RWT). LV systolic function was assessed using the LV ejection fraction (LVEF), which is obtained using the biplane method of discs from the apical four- and two-chamber views, according to the modified biplane Simpson’s method^14^. The left atrial (LA) volume was assessed using the Cube method^15^ and indexed to the body surface area. The peak early diastolic tissue velocity (e′) was measured from the septal aspect of the mitral annulus. Mitral inflow velocity was assessed using pulsed-wave Doppler from the apical four-chamber view, and the peak tricuspid regurgitation (TR) velocity was measured. Diastolic function was evaluated based on the following four criteria: septal E/e′ ≤ 14; septal e′ ≥ 7 cm/s; TR velocity ≤ 2.8 m/s; and LA volume index ≤ 34 mL/cm^2^. If > 50% of these criteria were met, then the participant was classified as having normal diastolic function; otherwise, the participant was classified as having abnormal diastolic function^13^.

### Clinical outcomes

The primary outcome was the incidence of MACE, including death, hospitalization because of acute coronary syndrome (ACS), any percutaneous coronary intervention (PCI), or hospitalization for HF. ACS was defined as the development of ischemic angina symptoms (≥ 10 min in duration) accompanied by changes on 12-lead electrocardiography or increased cardiac-specific biomarker levels. Any PCI was defined as the need for clinically driven revascularization that occurred following discharge from index hospitalization, as per the Academic Research Consortium definitions. Hospitalization for HF was defined as hospitalization wherein the patient exhibited signs and symptoms of HF on admission and was treated for HF during admission with medications, including diuretic therapy (either intravenous diuretics or augmentation of oral diuretics), vasodilators, inotropic support, or ultrafiltration.

### Statistical analysis

Data presented as frequencies (percentages) were assessed using the chi-squared or Fisher’s exact test for categorical variables, and those presented as mean ± standard deviation were assessed using the independent two-sample t-tests or Mann–Whitney tests for continuous variables. The follow-up period was analyzed using the median and interquartile range (IQR), with the 1st quartile and 3rd quartile values provided to describe the distribution of the follow-up times. A Kolmogorov–Smirnov test confirmed the non-normal distribution, supporting the use of these descriptive statistics. Despite the general guideline recommending a maximum of 4–5 independent variables based on the event per variable criterion of 10, given the total count of 48 MACE in our study, our research team chose to include a total of 8 variables. These were selected based on their significance in baseline characteristics and their known association with cardiovascular risks—specifically, age, hypertension, diabetes mellitus, dyslipidemia, current smoker status, the presence of obstructive CAD as determined by coronary angiography, and abnormal LV geometry and diastolic function as assessed by echocardiography. We conducted two primary analyses to explore the relationships of interest. First, to determine the association between abnormal diastolic function and other variables, both univariable and multivariable logistic regression analyses were carried out. The final multivariable logistic model was then constructed using backward elimination to identify the best Akaike’s information criterion; the odds ratios (OR) and 95% confidence intervals (CI) were identified. Second, to examine the relationship between these variables and MACE, we employed univariable and multivariable Cox regression analyses. In order to maintain consistency and rigor in our investigation, we used the same set of variables across both analyses. To compare MACE according to the menopausal age and diastolic function, we constructed Kaplan–Meier curves of MACE and composite outcomes for the groups with early and normal menopause.

Additionally, we conducted an analysis that employed inverse probability weighting (IPW) to adjust for the effects of confounding factors. This entailed estimating the inverse of the propensity scores for each group. Post-IPW, the standardized mean differences for all matched covariates were maintained within a 10% range, indicating effective balance. All analyses were two-tailed, and a *p*-value of < 0.05 was considered statistically significant. All data analyses were conducted using R software version 4.2.1 (R Core Team, Vienna, Austria).

## Results

**Figure 1:**
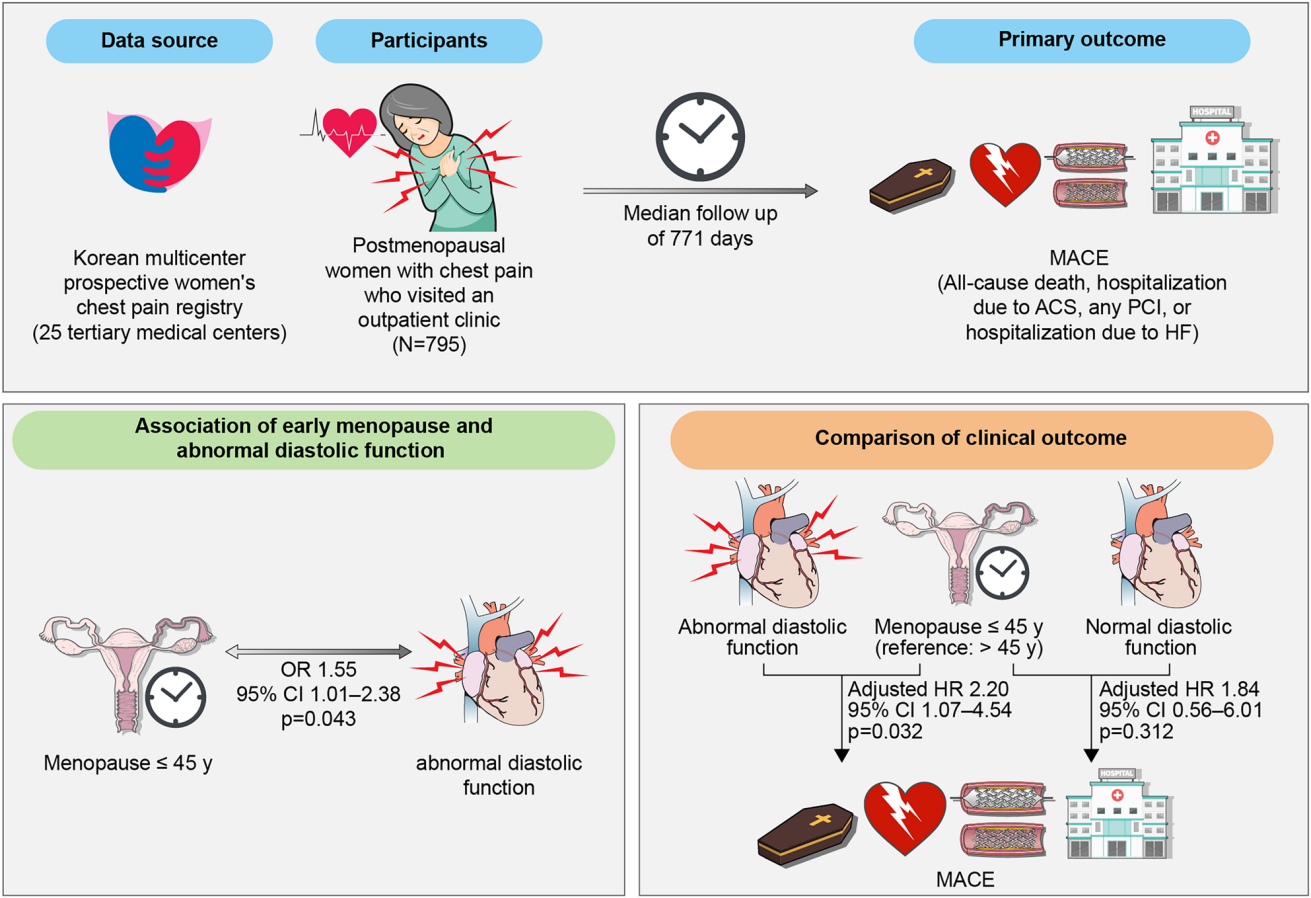
Central illustration. Analysis of data from the Korean multicenter prospective women’s chest pain registry, involving 795 postmenopausal women with chest pain who visited an outpatient clinic, revealed that women with a history of early menopausal age are associated with abnormal diastolic function and consequently demonstrate an increased risk of major adverse cardiovascular events. ACS, acute coronary syndrome; CI, confidence interval; HR, hazard ratio; MACE, major adverse cardiovascular events; PCI, percutaneous coronary intervention.

The overall study design and results are illustrated in Fig. 1.

### Baseline characteristics

**Figure 2:**
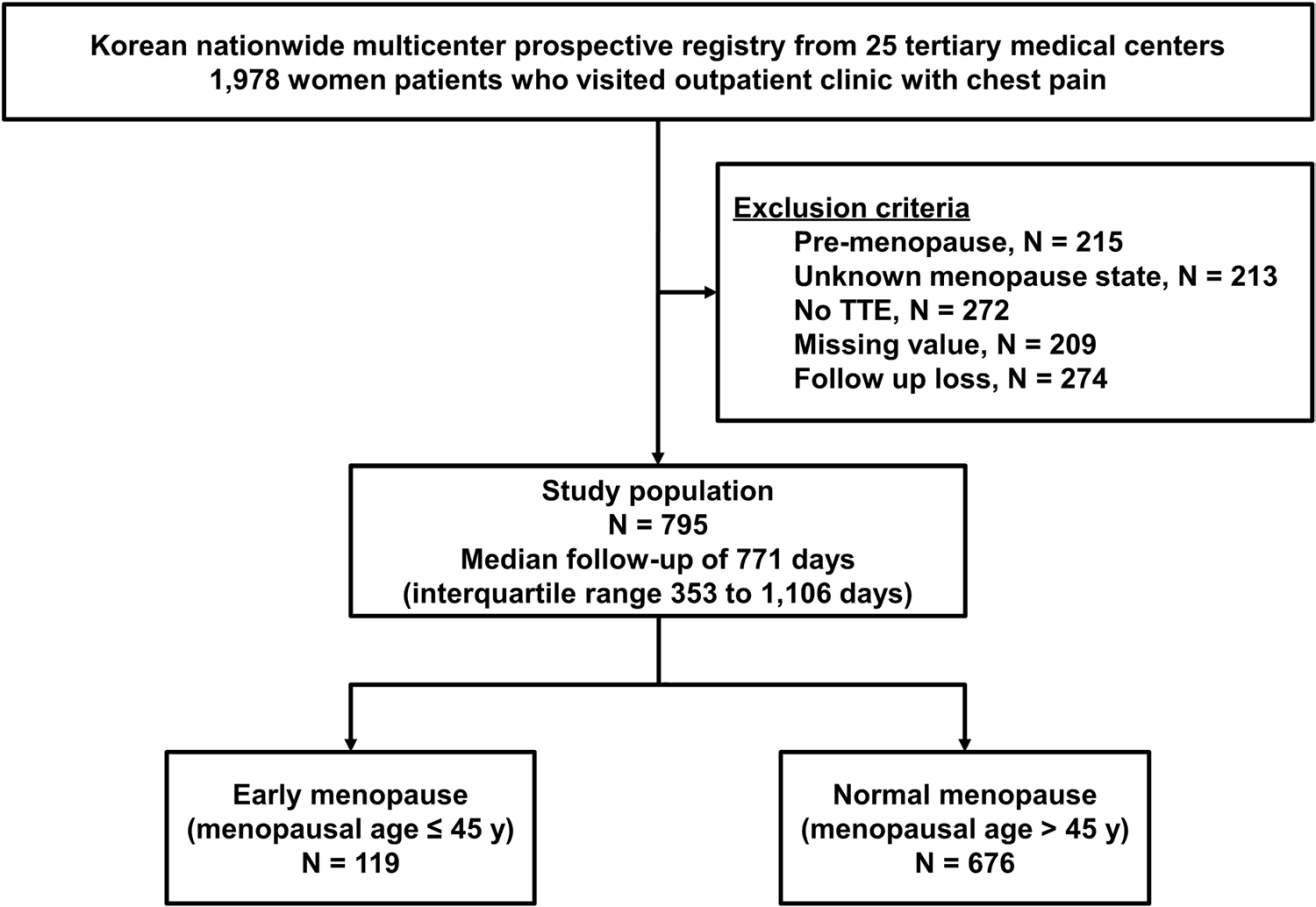
Study flowchart. KoROSE, Korean Women’s Chest Pain Registry; TTE, transthoracic echocardiography.

From the initial 1,978 patients, our analysis included 795 menopausal women (early menopause: 119 [15.0%]); normal menopause: 676 [85.0%]), after excluding 215 pre-menopausal patients, 213 with unknown menopausal status, 272 without TTE data, 209 with missing values, and 274 lost to follow-up (Fig. 2). The average age was 65.1 ± 8.6 years, and body mass index did not differ significantly between the two groups. However, the waist circumference was significantly higher in the early menopause group than in the normal menopause group (84.1 ± 9.2 vs. 80.8 ± 8.3 cm, *p* = 0.001) (Table 1). The early menopause group reported a higher prevalence of squeezing or pressure pain (75.6% vs. 60.1%, *p* = 0.002) and precordial or retrosternal area pain (80.7% vs. 69.7%, *p* = 0.008). The prevalence of DM was significantly higher in the early menopause group than in the normal menopause group (35.3% vs. 22.2%, *p* = 0.003). Regarding women's reproductive histories, the average menopausal age was 51.6 ± 5.6 years, and the reproductive life span was significantly shorter in the early menopause group than in the normal menopause group (26.3 ± 4.6 vs. 37.4 ± 4.2 years, *p* < 0.001). Medication history did not differ between the two groups, except hormonal replacement therapy (HRT) (18.5% vs. 9.8%, *p* = 0.008).

**Table 1:** Baseline characteristics.

	Overall (N = 795)	Early menopause (N = 119)	Normal menopause (N = 676)	*p*
Clinical characteristics				
Age, mean ± SD, years	65.1 ± 8.6	64.7 ± 9.6	65.2 ± 8.4	0.525
Height, mean ± SD, cm	154.7 ± 6.6	154.3 ± 6.4	154.7 ± 6.6	0.548
Weight, mean ± SD, kg	60.8 ± 9.7	61.8 ± 12.6	60.6 ± 9.1	0.342
BMI, mean ± SD, kg/m^2^	25.4 ± 3.5	25.9 ± 4.1	25.3 ± 3.4	0.200
Waist circumference, mean ± SD, cm	81.6 ± 8.6	84.1 ± 9.2	80.8 ± 8.3	0.001
Systolic BP, mean ± SD, mmHg	131.2 ± 18.6	131.5 ± 20.1	131.2 ± 18.2	0.877
Diastolic BP, mean ± SD, mmHg	76.8 ± 11.9	77.3 ± 11.5	76.7 ± 12.0	0.653
Heart rate, mean ± SD, bpm	74.0 ± 12.7	75.0 ± 11.7	73.8 ± 12.9	0.488
Cardiovascular risk factor				
Hypertension, number (%)	538 (67.7%)	90 (75.6%)	448 (66.3%)	0.057
Diabetes mellitus, number (%)	192 (24.2%)	42 (35.3%)	150 (22.2%)	0.003
Dyslipidemia, number (%)	478 (60.1%)	80 (67.2%)	398 (58.9%)	0.106
Current smoker, number (%)	41 (5.2%)	6 (5.0%)	35 (5.2%)	1.000
Women’s reproductive history				
Menopause age, mean ± SD, years	51.6 ± 5.6	41.9 ± 3.6	53.3 ± 3.8	< 0.001
Menarche, mean ± SD, years	15.9 ± 1.9	15.6 ± 2.2	15.9 ± 1.9	0.147
Reproductive life span, mean ± SD, years	35.8 ± 5.8	26.3 ± 4.6	37.4 ± 4.2	< 0.001
Medication history				
Antiplatelet medication				
Aspirin, number (%)	367 (46.2%)	61 (51.3%)	306 (45.3%)	0.267
Clopidogrel, number (%)	236 (29.7%)	38 (31.9%)	198 (29.3%)	0.636
Hypertensive medication				
RAAS inhibitor, number (%)	262 (33.0%)	44 (37.0%)	218 (32.2%)	0.365
Beta blocker, number (%)	279 (35.1%)	47 (39.5%)	232 (34.3%)	0.324
Calcium channel blocker, number (%)	228 (28.7%)	35 (29.4%)	193 (28.6%)	0.935
Diuretics, number (%)	68 (8.6%)	10 (8.4%)	58 (8.6%)	1.000
Antidiabetic medication, number (%)	136 (17.1%)	23 (19.3%)	113 (16.7%)	0.572
Anti-dyslipidemia medication, number (%)	426 (53.6%)	72 (60.5%)	354 (52.4%)	0.123
HRT, number (%)	88 (11.1%)	22 (18.5%)	66 (9.8%)	0.008

BMI, body mass index; BP, blood pressure; HRT, hormonal replacement therapy; RAAS, Renin–angiotensin–aldosterone system.

### Angiographic and echocardiographic characteristics

Early menopause was associated with a higher prevalence of obstructive CAD (48.7% vs. 34.8%, *p* = 0.005) and multivessel disease (*p* = 0.004) than was normal menopause. (Supplementary Table 1). The LV systolic function and regional wall motion abnormality index did not differ significantly between the two groups. However, the incidence of abnormal LV geometry and abnormal diastolic function was significantly higher in the early menopause group than in the normal menopause group (abnormal LV geometry, 61.3% vs. 50.8%, *p* = 0.022; abnormal diastolic function, 43.7% vs. 29.6%, *p* = 0.003).

### Association between clinical variables and abnormal diastolic function

Multivariable logistic regression analysis based on baseline, angiographic, and transthoracic echocardiographic characteristics revealed that age ≥ 65 years (OR 2.16; 95% CI 1.53–3.04; *p* < 0.001), hypertension (OR 1.54; 95% CI 1.06–2.23; *p* = 0.023), diabetes (OR 1.96; 95% CI 1.37–2.80; *p* = 0.001), obstructive CAD (OR 1.55; 95% CI 1.11–2.16; *p* = 0.009), abnormal LV geometry (OR 1.87; 95% CI 1.34–2.61; *p* = 0.001), and early menopause (OR 1.55; 95% CI 1.01–2.38; *p* = 0.043) were associated with abnormal diastolic function (Table 2).

**Table 2:** Impact of clinical variables, including early menopause, on abnormal diastolic function: insights from univariable and multivariable logistic regression analyses.

	Univariable analysis			Multivariable analysis		
	Odds ratio	95% CI	*p*	Odds ratio	95% CI	*p*
Age ≥ 65 years	2.97	2.15–4.11	< 0.001	2.16	1.53–3.04	< 0.001
Hypertension	2.19	1.55–3.10	< 0.001	1.54	1.06–2.23	0.023
Diabetes	2.62	1.87–3.67	< 0.001	1.96	1.37–2.80	0.001
Dyslipidemia	1.26	0.93–1.72	0.140			
Current smoker	1.40	0.74–2.68	0.303			
Obstructive CAD	2.25	1.66–3.06	< 0.001	1.55	1.11–2.16	0.009
Abnormal LV geometry	2.63	1.91–3.60	< 0.001	1.87	1.34–2.61	0.001
Early menopause	1.85	1.24–2.75	0.002	1.55	1.01–2.38	0.043

CAD, coronary artery disease; CI, confidence interval; LV, left ventricle.

### Clinical outcomes

**Figure 3:**
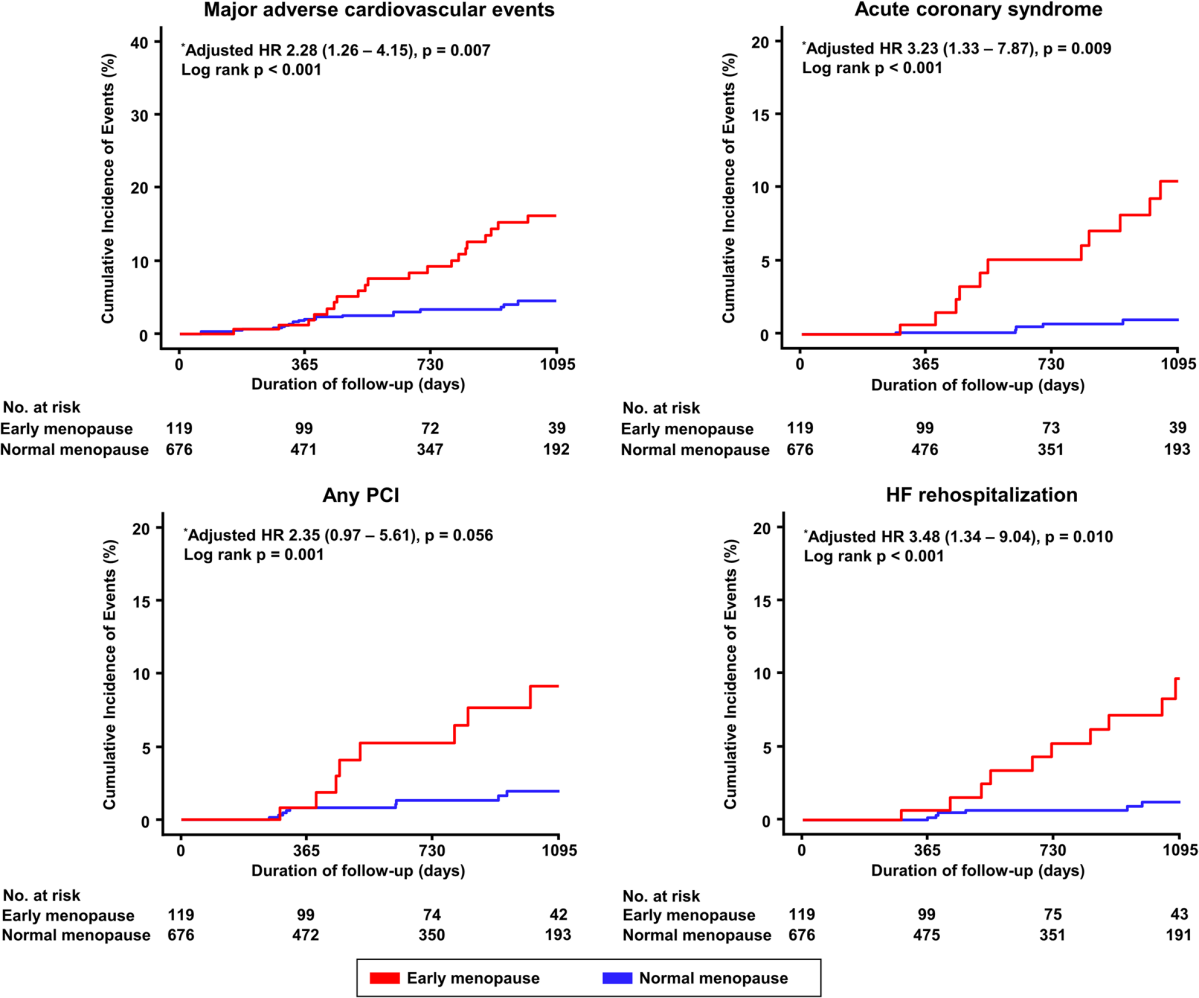
Cumulative incidence of clinical outcomes. HF, heart failure; HR, hazard ratio; PCI, percutaneous coronary intervention. *Adjusted variables: age ≥ 65 years, hypertension, diabetes, dyslipidemia, current smoker, obstructive CAD, abnormal LV geometry, and abnormal diastolic function.

**Figure 4:**
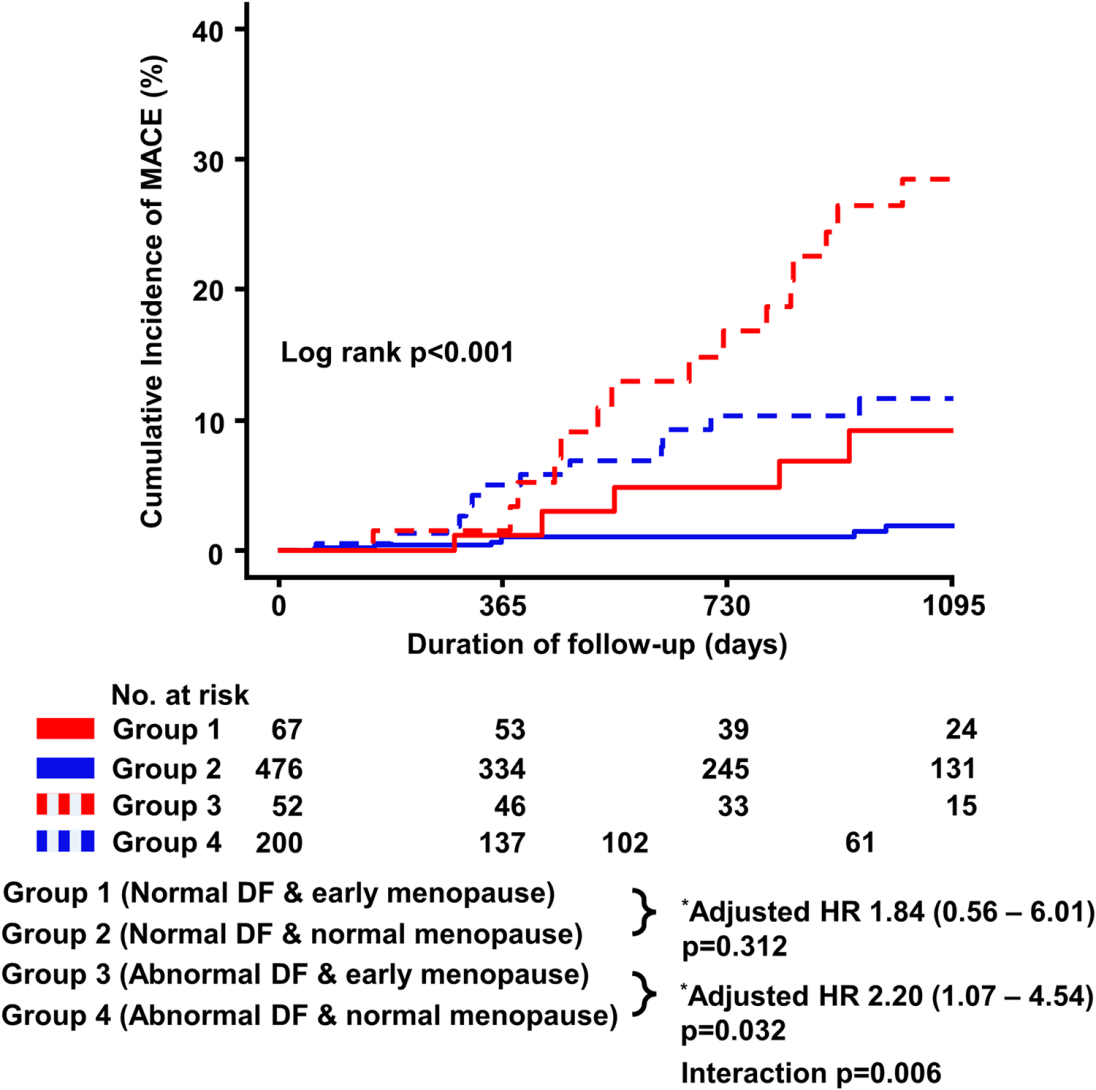
Cumulative incidence of MACE stratified by diastolic function and menopausal age. DF, diastolic function; HR, hazard ratio; MACE, major adverse cardiovascular events. ^*^Adjusted variables: age ≥ 65 years, hypertension, diabetes, dyslipidemia, current smoking, obstructive CAD, and abnormal LV geometry.

During a median follow-up period of 771 days (IQR 353–1.106 days), the risk of MACE was significantly higher in the early menopause group than in the normal menopause group (17.6% vs. 4.0%; adjusted-hazard ratio [HR], 2.28; 95% CI 1.26–4.15; *p* = 0.007) (Table 3). Regarding individual clinical outcomes, compared with normal menopause, early menopause was significantly correlated with an increased incidence of ACS (adjusted-HR 3.23; 95% CI 1.33–7.87; *p* = 0.009) and hospitalization for HF (adjusted-HR 3.48; 95% CI 1.34–9.04, *p *= 0.01) (Fig. 3). These results were consistent with those obtained after accounting for IPW-adjusted baseline characteristics and confounding factors (Supplemental Fig. 2). In patients with abnormal diastolic function, the risk of MACE was significantly higher in the early menopause group than in the normal menopause group (adjusted-HR 2.20; 95% CI 1.07–4.54; *p* = 0.032) (Fig. 4). However, no significant difference was found between the two groups for patients with normal diastolic function (adjusted-HR 1.84; 95% CI 0.56–6.01; *p* = 0.312), but the interaction between menopausal age and clinical outcomes was significant (*p*-value for interaction = 0.006).

**Table 3:** Risk of MACE between early menopause and normal menopause.

	Early menopause (N = 119)	Normal menopause (N = 676)	Unadjusted		Adjusted^b^		IPW-adjusted	
			HR (95% CI)	*p*	HR (95% CI)	*p*	HR (95% CI)	*p*
MACE^a^, number (%)	21 (17.6%)	27 (4.0%)	3.43 (1.93–6.11)	< 0.001	2.28 (1.26–4.15)	0.007	2.68 (1.34 – 5.36)	0.005
All-cause death, number (%)	4 (3.4%)	6 (0.9%)	3.32 (0.94–11.8)	0.063	2.66 (0.70–10.1)	0.153	2.30 (0.48 – 1.09)	0.296
ACS, number (%)	12 (10.1%)	10 (1.5%)	4.86 (2.07–11.4)	< 0.001	3.23 (1.33–7.87)	0.009	3.35 (1.25 – 8.94)	0.016
Any PCI, number (%)	11 (9.2%)	11 (1.6%)	4.18 (1.80–9.74)	< 0.001	2.35 (0.97–5.61)	0.056	2.66 (0.87 – 8.16)	0.086
Hospitalization of HF, number (%)	11 (9.2%)	8 (1.2%)	5.78 (2.31–14.5)	< 0.001	3.48 (1.34–9.04)	0.010	3.75 (1.26 – 11.2)	0.018

^a^MACE was defined as a composite of all-cause death, ACS, any PCI, or hospitalization for HF.

^b^Adjusted variables: age ≥ 65 years, hypertension, diabetes, dyslipidemia, current smoking, obstructive CAD, abnormal LV geometry, and abnormal diastolic function.

ACS, acute coronary syndrome; CI, confidence interval; HR, hazard ratio; IPW, inverse probability weight; LV, left ventricular; MACE, major adverse cardiovascular events; CAD, coronary artery disease.

## Discussion

In this study, we investigated the association between menopausal age and clinical outcomes in patients with intermediate CVD risk. We found that early menopause was associated with abnormal diastolic function (OR 1.55; 95% CI 1.01–2.38; *p* = 0.043) and that the risk of MACE was more than 4.4 times higher among women with early menopause (17.6% vs. 4.0%; adjusted-HR 2.28; 95% CI 1.26–4.15; *p* = 0.007), with persistent associations observed after adjusting for conventional CVD risk factors. Notably, patients with early menopause and abnormal diastolic function had a significantly higher risk of MACE than those with normal menopause; however, no significant difference was found between the two groups in patients with normal diastolic function.

Our study expands upon this understanding by elucidating the direct impact of early menopause on left ventricular diastolic dysfunction and its subsequent role in increasing the risk of cardiovascular events. Our findings are in line with the conclusions of significant studies by Muka et al., de Kleijn et al., and Zhu et al.^16–18^, which all highlight early menopause as a factor significantly associated with an increased risk of cardiovascular diseases, reinforcing the importance of menopausal timing on cardiovascular health outcomes. Furthermore, our findings add a new dimension to the existing literature by detailing the specific mechanisms through which early menopause exacerbates cardiovascular risk, thereby offering potential targets for early intervention.

Our study showed that early menopause was associated with a higher prevalence of DM, obstructive CAD, and multivessel disease. Several mechanisms have been proposed to explain the association between early menopause and poor clinical outcomes, with the most likely cause being the lack of estrogen. Estrogen is a potent vasoactive hormone that promotes vascular remodeling and elasticity and can regulate reactive dilation and local inflammatory activity^19^, as well as endothelial vasodilator dysfunction, in response to estrogen deficiency^20^. Estrogens also play a key role in regulating calcium homeostasis and, thus, in fine-tuning the normal process of cardiomyocyte contraction and relaxation^21^. Cho et al. showed that LV diastolic dysfunction was only associated with obstructive CAD in women^22^. In the Study of Women Across the Nation research, post-menopausal women had smaller high-density lipoprotein particles than did pre-menopausal women, suggesting that the protective effect of high-density lipoprotein cholesterol might be altered in post-menopausal women because of changes in the lipoprotein subclass profile^23^. Therefore, decreased estrogen levels over the menopause transition period may lead to atherosclerosis owing to endothelial vasodilator dysfunction and an altered lipoprotein profile, which may subsequently contribute to an increased CVD risk.

Menopause is characterized by physiological changes affecting several organs and systems, with the cardiovascular system being the most significantly affected^24^. Several studies have shown that menopause may be associated with impaired LV systolic and diastolic cardiac function^24,25^. Although early menopause is widely believed to be associated with a greater risk of cardiovascular events, its relationship with incident HF has not been investigated in detail. Our study demonstrated that early menopause was significantly associated with increased hospitalization for HF. Even after adjusting for baseline characteristics, the risk of MACE was significantly higher for those with early menopause who had abnormal diastolic function. However, no significant difference was found between the two groups among patients with normal diastolic function. Our study supports recent evidence showing that diastolic dysfunction is a critical predictor of long-term clinical outcomes, emphasizing its value in assessing patient prognosis^26^.

In our study, the LV systolic function and regional wall motion abnormality index did not differ between the two groups. However, early menopause was associated with significantly increased proportions of LV hypertrophy and abnormal diastolic function than those associated with normal menopause. Similar to myocardial hypertrophy, estrogen has been shown to modulate key mechanisms involved in diastolic function, including the regulation of calcium ion channel homeostasis and protein kinase A activity, which are important for myocyte relaxation^11^. Myocardial hypertrophy is one of the most common causes of abnormal diastolic function, both of which are inhibited by estrogen signaling and were found in the present study to progress in an adverse direction after menopause. Estrogen is a key modulator of the cyclic guanosine monophosphate pathway, which is an important stimulator of LV relaxation^27^. Women with HF have a smaller and stiffer LV than do men^28^. Our findings also revealed that women with early menopause had a significantly higher waist circumference compared to those with normal menopause (84.1 ± 9.2 cm vs. 80.8 ± 8.3 cm; *p* = 0.001). This observation is particularly concerning as higher waist circumference, indicative of increased visceral fat, has been linked to estrogen deficiency^29^. This is probably due to the direct effects of estrogen, which inhibits collagen production in female cardiac fibroblasts but stimulates it in men. In addition to changes in estrogen levels, menopause is also associated with an increase in visceral fat^30^, and both circulating adipokines and localized visceral fat depots have been associated with abnormal diastolic function and HFpEF^31^. The decrease in estrogen levels during early menopause may thus contribute to an increase in visceral fat, further exacerbating the risk of cardiovascular diseases by promoting a pro-inflammatory state and affecting lipid metabolism^32^. These biological pathways may be the underlying mechanisms occurring in early menopause through which abnormal diastolic function is associated with HF.

These findings have important clinical and public health implications. First, identifying women with early menopause offers a window of opportunity to implement the active management of other CVD risk factors to improve overall cardiovascular health in their post-menopausal years. These women may also require close clinical monitoring. Second, evaluating diastolic function through echocardiography in early menopausal patients plays an important role in predicting HF risk. To build upon the body of knowledge concerning early menopause and cardiovascular health, future studies are crucial. Further research is needed to evaluate prognostic differences according to female hormones and abnormal diastolic function in early menopause and to identify the gaps and directions for further research. Specifically, investigating whether improved diastolic function through targeted interventions can lead to improved prognosis in this population is of paramount importance.

This study had a few limitations. First, the age of menopause was calculated based on self-reports, which may have been flawed because of recall bias. However, previous studies have demonstrated good validity and reproducibility of self-reported age at menopause^33^. Validated questionnaires and standard questions were used in each of these previous studies; thus, we assumed that the heterogeneity of menopausal status among studies was limited. Second, detailed data regarding the age of DM diagnosis were not collected, which limits our ability to explore the potential impact of early diabetes onset on the timing of natural menopause. This omission restricts our understanding of how diabetes might influence menopausal age, a factor that has been shown to be significant in previous research^34,35^. Third, detailed data regarding the types or duration of menopausal hormone therapies (estrogen alone or estrogen with progestin) were not collected, underscoring a gap in our analysis that could have provided valuable insights into the cardiovascular health of menopausal women. Nevertheless, one study found a similar association between different types of menopausal hormone therapies (estrogen alone or estrogen with progestin) and the incidence of coronary heart disease or stroke^36^. Fourth, despite adjusting to minimize bias based on several sensitivity analyses for different baseline characteristics, it is possible that unmeasured confounders existed between the two groups. Therefore, we performed multivariable Cox regression and IPW-adjusted analyses to adjust for confounding factors as much as possible, which led to consistent results in this study. Fifth, our study’s exclusion criteria led to the omission of patients with conditions such as end-stage renal disease, malignancy, primary pulmonary hypertension, COPD, and autoimmune diseases, to ensure a focus on the specific impacts of early menopause and diastolic dysfunction on cardiovascular outcomes. However, the absence of specific exclusion counts in our dataset restricts our ability to quantify the direct impact of these conditions on our findings. This limitation may affect the generalizability of our results to broader populations with these comorbidities. Sixth, our study, which serves as a pioneering exploration of the association between early menopause, diastolic function, and MACE outcomes, did not undertake formal sample size calculations or power analyses due to its pilot nature. Moreover, our study design, influenced by the accessibility and availability of participants within our clinical setting, may not fully capture the diversity of the general population.

## Conclusion

This nationwide, multicenter, prospective study revealed that early menopause was associated with abnormal diastolic function regardless of the presence of obstructive CAD and showed a higher risk of MACE, ACS, and hospitalization for HF. These findings highlight early menopause and abnormal diastolic function as potential risk markers in women for CVD events in midlife.

### Supplementary Information

Supplementary Information.

## Data Availability

The datasets used and/or analyzed during the current study are available from the corresponding author on reasonable request.
