# Supplementary material for: Early menopause is associated with abnormal diastolic function and poor clinical outcomes in women with suspected angina
Source: Sci Rep. 2024 Mar 15;14:6306. doi: 10.1038/s41598-024-57058-2 (PMC10943187; doi:10.1038/s41598-024-57058-2)
Supplement: Supplementary file 1 — Supplementary Information. [file 41598_2024_57058_MOESM1_ESM.pdf]

Supplementary Appendix

Supplementary Table 1. Angiographic and transthoracic echocardiographic characteristics

Supplementary Figure 1. Questionnaires of patients with chest pain in KoROSE registry

Supplementary Figure 2. Covariate balance between early and normal menopause – inverse probability weighting

Supplemental Table 1. Angiographic and transthoracic echocardiographic characteristics

Variable	Total (N=795)	Early menopause (N=119)	Normal menopause (N=676)	p
Obstructive CAD, n (%)	293 (36.9%)	58 (48.7%)	235 (34.8%)	0.005
Severity of CAD				0.004
Normal, n (%)	502 (63.1%)	61 (51.3%)	441 (65.2%)	
One-vessel disease, n (%)	182 (22.9%)	30 (25.2%)	152 (22.5%)	
Two-vessels disease, n (%)	77 (9.7%)	21 (17.6%)	56 (8.3%)	
Three-vessels disease, n (%)	34 (4.3%)	7 (5.9%)	27 (4.0%)	
LV structure				
LVEDD, mean ± SD, cm	46.4 ± 4.6	47.3 ± 5.4	46.2 ± 4.4	0.053
LVESD, mean ± SD, cm	28.6 ± 5.3	29.9 ± 6.2	28.4 ± 5.1	0.018
Septal wall thickness, mean ± SD, cm	9.6 ± 1.7	9.8 ± 1.7	9.5 ± 1.6	0.166
Posterior wall thickness, mean ± SD, cm	9.2 ± 1.4	9.4 ± 1.5	9.2 ± 1.3	0.206
LV mass index, mean ± SD, g/m^2	93.9 ± 22.6	99.8 ± 26.2	92.8 ± 21.8	0.011
RWT, mean ± SD	0.40 ± 0.08	0.40 ± 0.09	0.40 ± 0.08	0.876
Abnormal LV geometry	410 (52.43%)	73 (61.34%)	337 (50.83%)	0.044
Concentric remodeling, n (%)	111 (14.2%)	13 (10.9%)	98 (14.8%)	
Concentric hypertrophy, n (%)	141 (18.0%)	25 (21.0%)	116 (17.5%)	
Eccentric hypertrophy, n (%)	158 (20.2%)	35 (29.4%)	123 (18.6%)	
LV systolic function				
Ejection fraction, mean ± SD, %	59.7 ± 8.1	60.2 ± 9.1	59.7 ± 7.9	0.522
WMSI, mean ± SD,	1.03 ± 0.13	1.02 ± 0.13	1.03 ± 0.13	0.724
LV diastolic function				
E/A ratio, mean ± SD	0.85 ± 0.46	0.84 ± 0.26	0.86 ± 0.48	0.729
Septal e’, mean ± SD, cm/s	6.04 ± 1.93	5.65 ± 1.80	6.11 ± 1.94	0.015
E/e’, mean ± SD	11.6 ± 4.3	13.1 ± 5.2	11.3 ± 4.1	0.001
LAVI, mean ± SD, cm^2/m^2	30.1 ± 22.8	31.7 ± 20.9	29.8 ± 23.1	0.428

Variable	Total (N=795)	Early menopause (N=119)	Normal menopause (N=676)	p
TR velocity, mean ± SD, m/s	2.38 ± 0.31	2.41 ± 0.32	2.37 ± 0.30	0.295
Classification of diastolic function				0.003
Normal diastolic function, n (%)	543 (68.3%)	67 (56.3%)	476 (70.4%)	
Abnormal diastolic function, n (%)	252 (31.7%)	52 (43.7%)	200 (29.6%)	

A wave, peak late diastolic velocity of mitral inflow; CAD, coronary artery disease; E wave, peak early diastolic velocity of mitral inflow; e' wave, peak early diastolic velocity of mitral septal annulus; LAVI, left atrial volume index; LV, left ventricle; LVEDD, left ventricular end-diastolic diameter; LVESD, left ventricular end-systolic diameter; PASP, pulmonary artery systolic pressure; TR, tricuspid regurgitation; WMSI, wall motion score index

Supplementary Figure 1. Questionnaires of patients with chest pain in KoROSE registry

Questionnaires of patients with chest pain in KoROSE registry

1. Patient Information

Age ______years

Date of birth ______Year ______Month ______Day

Body measurements Height ______cm, Weight ______kg, Waist circumference ______cm

2. Risk factors

DM ________ yr ☐Yes HbA1c ______ ☐No

Hypertension ________ yr ☐Yes ☐No

Smoking (Current smoking or quit within 1 month) ☐Yes ☐ less than 10 cigarett

☐ more than 10

☐No ☐ Ex smoker

Family Hx of coronary artery disease ☐Yes ☐No

Lipid level

Total cholesterol ______ mg/dl LDL colesterol______ mg/dl

HDL cholesterol ______ mg/dl Triglyceride ______ mg/dl

Serum creatinine ______ mg/dl

hsCRP ______ mg/L

3. Gynecological and Obsteric history

Menopause ☐ No

☐ Yes ---- what age ______yr

HRT ☐ Yes ----- how long ____ yr

☐No

Menarche ______ yr

How many pregnancy _________ times

History of pre/eclamsia ☐ Yes ☐ No

Twin pregnancy ☐ Yes ☐ No

POS ☐ Yes ☐ No

Marital status ☐ Single ☐ Married ☐ Divorced ☐ Widowed

Occupational history ☐ Production worker ☐ Office worker ☐ None or housewife

4. Current medication

☐ Antihypertensive Drugs
☐ Circulatory medications (aspirin, zincomine, omega-3)
☐ Diabetes medications
☐ Female hormone medications
☐ Thyroid medications
☐ Antidepressants
☐ Sleeping pills
☐ Anti-arthritis medications
☐ Chronic headache and chronic pain medications
☐ Gastrointestinal system drugs
☐ Herbal medicine
☐ Osteoporosis
☐ Vitamins and other dietary supplements

5. Physical activity

☐ I don't do anything but chores ☐ I stay active, even if I don't exercise
☐ irregular exercise
☐ Regular exercise
  ☐ Below 2hrs/ wks ☐ 2-4hrs/wks
  ☐ 4-6hrs/wks ☐ 6-10hrs/wks

6. Characteristics of chest pain

1) Character
☐ Sqeezing ☐ Pressure ☐ Stinging ☐ Burning ☐ Dull ☐ Indescribable

2) Location
☐ Left side of chest ☐ Right side of chest ☐ Epigastric ☐ Retrosternal

3) Duration
☐ <5 minutes ☐ 5-15 minutes ☐ 15-60 minutes ☐ >1 hour
☐ For various lengths of time

4) Aggravating factors
☐ None ☐ Exercise ☐ Meal ☐ Psychological stress ☐ Low temperature ☐ Alcohol ,other _________

5) Location of radiating pain
☐ Left arm or left shoulder ☐ Right arm or right shoulder ☐ Neck ☐ Back

6) Associated symptoms
☐ Palpitation ☐ Dizziness ☐ Syncope ☐ Dyspnea ☐ Headache

Supplemental Figure 2. Covariate balance between early and normal menopause – inverse probability weighting

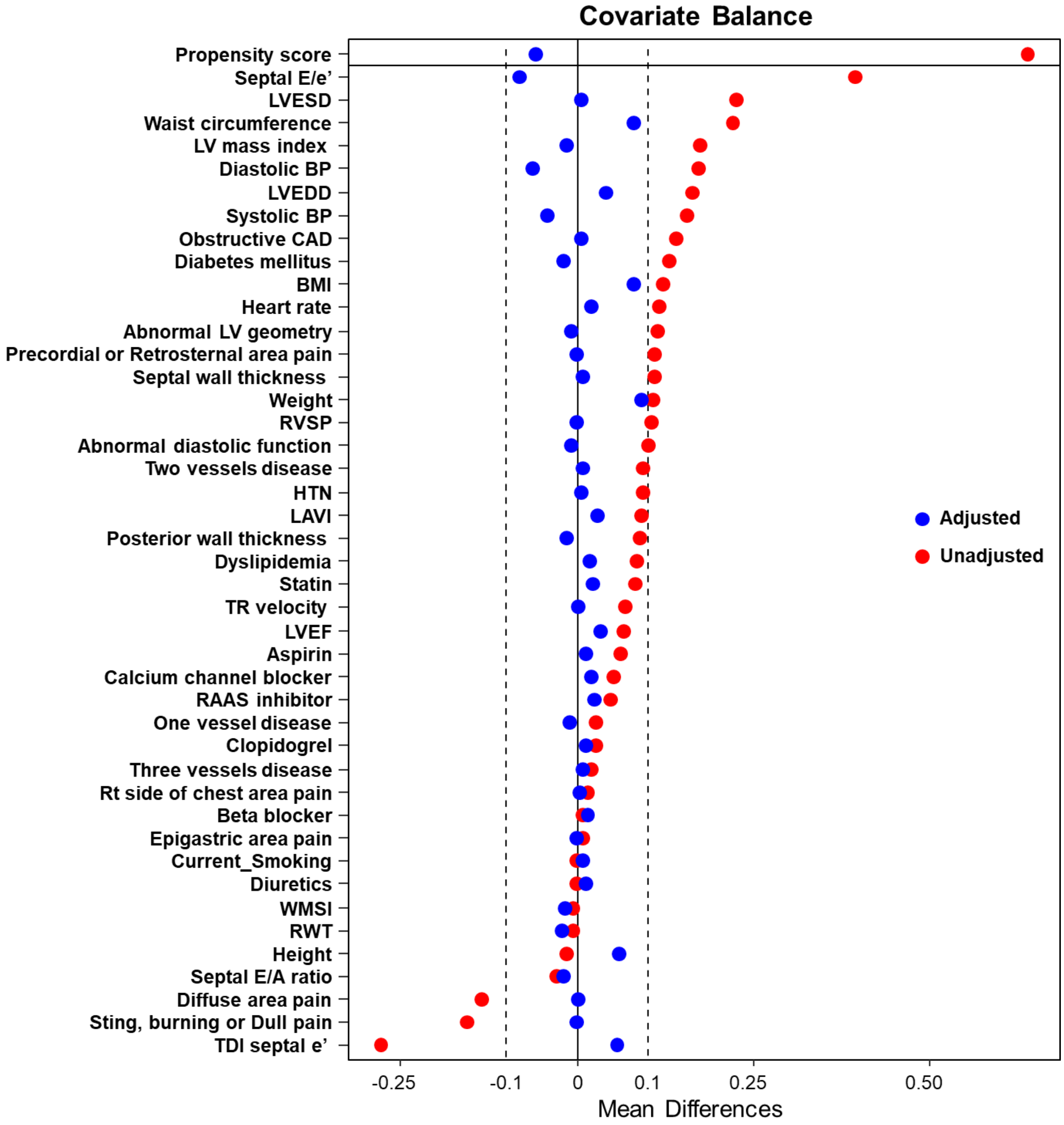

BMI, body mass index; BP, blood pressure; CAD, coronary artery disease; LAVI, left atrial volume index; LV, left ventricle; LVEDD, left ventricular end-diastolic diameter; LVESD, left ventricular end-systolic diameter; PASP, pulmonary artery systolic pressure; RAAS, Renin–angiotensin–aldosterone system; RVSP, right ventricular systolic pressure; TR, tricuspid regurgitation; WMSI, wall motion score index
